# Editorial: Mitochondrial Communication in Physiology, Disease and Aging

**DOI:** 10.3389/fcell.2019.00054

**Published:** 2019-04-11

**Authors:** Nuno Raimundo, Anita Krisko

**Affiliations:** ^1^Institute for Cellular Biochemistry, University Medical Center Göttingen, Göttingen, Germany; ^2^Mediterranean Institute for Life Sciences, Split, Croatia

**Keywords:** mitochondria, aging, age-related disease, lysosome, endoplasmic reticulum

Mitochondria hold the key to many biological functions, ranging from the long-known role in ATP production to metabolic control, cellular signaling and regulation of cell death (Raimundo, 2014). Many of these functions rely on complex signaling pathways elicited by perturbations of diverse aspects of mitochondrial function (Raimundo, 2014; Shadel and Horvath, 2015). Notably, it has recently started to be unveiled, that mitochondrial functions are supported by their communication with other cellular organelles (e.g., endoplasmic reticulum, lysosomes) and processes (e.g., autophagy, senescence).

In general, organelle communication can be manifested by the formation of direct contact sites via membrane apposition, as well as via signals released by one organelle that trigger a signaling pathway regulating the function or homeostasis of another organelle (Diogo et al., 2018). The research topic “Mitochondrial communication in physiology, disease and aging” (2017–2018) brought together an ensemble of perspectives on how mitochondria communicate with the intra and extracellular surroundings, in different model organisms. In this context, Pon and colleagues address the role of interorganellar mitochondrial tethers in aging using budding yeast as a model (Pernice et al.). Mitochondrial tethers are critical for non-uniform segregation of mitochondria during asymmetrical cell division in yeast, allowing the daughter cell to inherit fitter, and the mother to retain high-functioning mitochondria. It is possible that other cell types characterized by asymmetrical cell division, like human mammary stem-like cells, may display similar tether-dependent mechanisms of mitochondrial segregation.

The best characterized mitochondrial interaction is the one involving the contact sites between mitochondria and the endoplasmic reticulum (ER) (Cohen et al., 2018). These structures are conserved from yeast to humans, and they are important for the transfer of Ca^2+^ and lipids between the two organelles (Eisenberg-Bord et al., 2016). While many proteins that form the mitochondria-ER tethers have been identified, less is known about how the assembly or maintenance of these tethers is regulated upon stimulation. Giacomello and colleagues review how proteins are recruited to the mitochondria-ER contact sites and discuss their physiological role in these interfaces, as well as their role in stress signaling (Ilacqua et al.).

As mentioned above, there are interactions between mitochondria and ER that do not rely on contact sites. One such example is the activation of the mitochondrial unfolded protein response (UPRmt) in response to mitochondrial stress, as reviewed by Callegari and Dennerlein. Furthermore, this pathway is part of broader mitochondria-ER interaction spectrum. Mitochondria and ER both respond to changes in proteostasis status in each other cellular compartment resulting in a cell-wide response to compartmentalized proteostasis failure or improvement (Perić et al., 2016). Interestingly, this process relies on the crosstalk between Hsp82 and TORC1 complex, providing another example of communication between pathways in regulation of a cell homeostasis (Perić et al., 2017).

Another organelle interaction that has lately gathered attention is the mitochondria-lysosome crosstalk. Similar to the case of ER, contact sites between mitochondria and lysosomes have been described both in lower (Elbaz-Alon et al., 2015) and higher eukaryotes (Wong et al., 2018; Cioni et al., 2019). Germain and colleagues present a comprehensive discussion on the tethers between mitochondria and lysosomes as well as on the metabolic and signaling crosstalk between the two organelles (Todkar et al.). This question is further addressed by Mittelbrun and colleagues, who also consider the endosomal interactions with mitochondria and their implications for extracellular signaling (Soto-Heredero et al.). It is noteworthy to point out that acute and chronic defects in mitochondria have opposite regulatory effects on lysosomal biogenesis and function. While under acute mitochondrial stress activation of TFEB/MITF-dependent lysosomal (and, likely, mitochondrial) biogenesis is observed, chronic mitochondrial stress results in repression of both lysosomal function and biogenesis, resulting in the cytoplasmic accumulation of dysfunctional lysosomes with decreased hydrolytic capacity (Fernández-Mosquera et al., 2017; Fernandez-Mosquera et al., 2019). The role of mitochondria-lysosome crosstalk is also considered in the context of diseases. Plotegher and Duchen explore the impact of this organelle “duo” in Parkinson's disease (Plotegher and Duchen), while Fernandez-Checa and colleagues comprehensively integrate the mitochondria-lysosome crosstalk in the pathology of a lysosomal storage disease (Torres et al.). In both of these cases, primary defects in lysosomes result in perturbation of mitochondrial homeostasis and function. While this is often entirely attributed to the impairment of the autophagic pathway, new findings revealed the existence of a signaling pathway triggered in lysosomal sphingolipidosis (storage diseases of the lysosomal catabolism pathway), which results in the repression of mitochondrial biogenesis (Yambire et al., 2019). Moreover, one natural consequence of lysosomal impairment is the perturbation of the autophagy pathway, with consequent decrease in mitophagy. The vast majority of the studies in autophagy and mitophagy have been carried out in cultured cells, while gathering *in vivo* data from model organisms, particularly mammals, has been hampered by a lack of appropriate tools. In this research topic, Poulton and colleagues presented a novel tool to assess mitophagy in mice, and applied it to a mouse model of a mitochondrial disease, namely autosomal dominant optic atrophy (Diot et al.).

Finally, several signaling pathways that are active upstream and downstream of mitochondria were also addressed in this research topic, in multiple model organisms. For example, this includes the role of mitochondrial retrograde response in induction of yeast filamentous growth in a conditioned environment (Gonzalez et al.). On the other hand, using mouse embryonic fibroblast cell lines, presenilin-2, a protein involved in Alzheimer's disease (AD), is shown to have a role in the regulation of mitochondrial function in the context of the electron transport chain maintenance (Contino et al.). These results may contribute to the research of AD pathology, in particular of the AD-related metabolic decline. Lastly, an important role of the mitochondrial inner membrane as an independent signaling platform is carefully dissected by Dudek.

Altogether, this research topic has integrated mitochondrial function into a complex but comprehensive network with other cellular organelles and processes. While this view is evolving, due to the increasing attention that this field is awarded, it provides a time-stamp on how mitochondrial communication in physiology, pathology and aging is seen in 2018.

## Author Contributions

All authors listed have made a substantial, direct and intellectual contribution to the work, and approved it for publication.

### Conflict of Interest Statement

The authors declare that the research was conducted in the absence of any commercial or financial relationships that could be construed as a potential conflict of interest.
